# Antiseptics and Disinfectants

**Published:** 1884-10

**Authors:** E. J. Lilly

**Affiliations:** Circleville, O.


ARTICLE V.

### ANTISEPTICS AND DISINFECTANTS.

BY E. J. LILLY, M. D., CIRCLEVILLE, O.

[Written for the Mad River Valley Dental Society.]

So much has been said of late about antiseptics and disinfectants that seemingly nothing more remains to be disclosed. But in looking up the literature of the subject we find so many contradictory statements that it would almost be an impossibility, without direct experiment in each case, to tell what is true and what is not true. To carry out such a series of experiments, however, would require no little time and labor; for to gain any reliable results the conditions should be as diverse and varied as possible, the agents used in all degrees of strength, and the results, in every case, noted to the minutest particular. We haven't much that is new to offer in this regard, but will attempt a short sketch of what has been accomplished and is best known relative to the subject.

By an antiseptic we understand a substance which has the power of preventing decomposition in dead animal or vegetable matter. A certain degree of warmth, together with moisture and access of air, are necessary to the occurrence of putrefactive changes, which consist essentially in the breaking up of the complex organic compounds, and the formation of new and simpler combinations. During the process various gases are envolved, and lower forms of animal and vegetable life are observed to grow and multiply in the decomposing mass. Putrefaction may be prevented by removing one or more of the conditions under which it takes place. If, after subjecting the substance to a certain degree of heat, air (and of course the organisms suspended therein) be excluded, and dead matter can be preserved for an indefinite length of time. Extreme cold will accomplish the same result, though in a different way. Numerous chemical substances have the power of preventing decomposition, while others have a deodorizing property, acting by oxidizing, deoxidizing, or otherwise changing the chemical constitution of the substances, and are, in virtue of these properties, effective disinfectants to that degree.

A disinfectant proper is understood to be a substance, or preparation, that will not only arrest putrefaction by destroying the cause, but will, at the same time, render the products thereof harmless. There is no connection between the power which prevents decay from setting in and that which checks or stops it when once begun, for antiseptics in general have but a slight effect upon bacteria. Exceptions are found, of course, in those substances, like corrosive sublimate, which are violent poisons, and which are both antiseptic and disinfectant.

The increased attention bestowed, of late years, upon sanitary matters has led to the manufacture of numerous materials claiming to be valuable as antiseptics and disinfectants. But so many of them having been tried and found wanting, the tendency now is to undervalue and decry the use of nearly all. The failure to obtain satisfactory results is, in many instances, probably due to confounding antiseptics, with disinfectants and deodorizers, trying to make one agent do the work of another, and from ignorance, perfectly justifiable in many cases, of how, when, and where to apply them. For instance, if we wish to purify some water, in which is suspended decaying vegetable matter. What agent will best serve the purpose? Shall we use carbolic acid? or corrosive sublimate? The odor of the first would simply mask the other smell by its own, while the second would not even do that. Of course the mercuric chloride would be death to the animalcules in the water, but citric acid will detroy all of them, except cyclops and those with thick epidermis, in two minutes, and still leave the water fit for use. Evidently some oxidizing agent, like potassium permanganate, or, better still, hydrogen peroxide, is indicated. Again, we wish to be relieved of some obnoxious odors, of which hydrogen sulphide is the most prominent and disagreeable, and the question is, what agent to use in this case. Would creosote, salicylic acid, sodium chloride, or potassic permanganate answer? Neither of them would be of much value, but plumbic acetate, or some other metallic salt that would decompose and unite with the sulphide, would be the correct remedy. Or, take some putrefying animal matter. Plumbic acetate, to combine with the sulphur compounds, is indicated for one thing. But since, for the purpose of disinfection, it is not only necessary to destroy odors and retard the development of spores, but it is also needful to kill the micro-organisms, and that, too, in the shortest possible period. Therefore only such agents as are bacteriacides, like mercuric chloride, bromine, etc., can be effectively used in such a case, and are the agents to select.

The study and correct application of true antiseptics and disinfectants may be regarded as a very important practical question; for the sanitary weal of the individual, of cities, and even of countries, sometimes depends upon rational disinfection. But with even our most powerful agents, it is almost impossible, at times, to render innocuous the gases of decomposition and decay, or to accomplish the destruction of those living organisms of simple structure, whose importance relative to the propagation of disease we have so much reason for believing to exist, so tenacious, of life, so minute and unfindable are they. If we knew always just where to look for them, and, after having found them, what agent to apply to each species, what sized dose it was necessary to administer, etc., we could most assuredly contend against them successfully. As it is we are in the predicament the Irishman was with his flea remedy He had his brick all ready, but the flea had first to be caught before he could mash him with it.

Fortunately many of these noxious substances give us warning of their presence though the medium of the olfactory organs. It will be remembered that the sense of smell is excited by gases only, including of course under this term the vapor of liquids and solids which have low vapor-tension, and which, in consequence, give off vapors at the ordinary temperature. The difference in smells or odors is caused by the difference in the nature of the vibrations of the gaseous particles, just as the difference in the tone of musical sounds depends on the rate and on the nature of the vibrations which strike the tympanum. So we cannot properly say we smell a decomposing mass, but only the gases envolved during the process ; and we are too apt to think that the only deleterious products of decomposition are those that offend the nostrils, being satisfied when the odor has been destroyed. This is a mistake. Among the products of putrefaction are some gases, mostly sulphur compounds, of very repulsive odor. This is a most fortunate circumstances in one respect, as the odor not only serves to make its presence known, but prompts, the individual either to flee from it, or employ ventilation for comfort's sake. But it is not only foul-smelling substances that are harmful, for they may be deodorized yet retain their poisonous properties long after the odor is gone. For example, carbon disulphide, a very poisonous liquid, the vapors of which produce intense headache and vertigo, has, usually, a repulsive odor ; but it is not difficult to deodorize it without affecting its other properties. Deodorized alcohol is no less a poison than it was before. Then again there are substances, like carbon monoxide. marsh gas, etc., which possess no odor, and their presence is made manifest only by the effect produced in inhaling them. Against such substances we have no protection except nature's disinfectant, the atmosphere. Yet there are numbers of agents which, used intelligently, will bring about excellent results, and it will be found that each possesses a definite scope of action. Thus carbolic acid is, in general, the antiseptic for crude masses of matter, salicylic acid for the laboratory, while thymol, hydrogen peroxide, glycero-borates of sodium and potassium, listerine, etc., are to be used in the office.

There are so many materials claiming recognition as good antiseptics or disinfectants that to give a list of them would consume too much time ; but they all produce their effect by acting as poisons to the infusorial organisms which are held to be essential to the occurrence of putrefaction. A solution of quinine will prevent decomposition in an organic broth, as will also carbolic acid, salicylic acid, borax, alum, iron salts, etc., but they cannot properly be called disinfectants, although they are frequently used for the latter purpose. But carbolic acid, the essential oils, burnt coffee, scorched rags, and the like, all have an odor of their own that only masks the other smell without destroying it.

A very good disinfectant, and one that has become quite prominent of late, is listerine. Of course great things are claimed for, but only time and use can determine its true value. Peroxide of hydrogen is another agent that is extensively used for both its antiseptics and disinfectant properties. It is said to instantly arrest fermentations due to the development of living organisms, and the putrefaction of all materials which do not decomposed it. But, as it is rapidly destroyed by so many substances, its application would seem to be limited. When diluted with water and used in connection with prepared chalk, it is excellent as a dentifrice. A good preparation, combining antiseptic properties with a perfume, to drop into our cuspadors after cleansing, is the following:

R

| | |
|---|---|
| Eu de cologn - - - - - - | 8 fl. oz. |
| Peroxide of hydrogen - - - - | 2 drachm. |
| Chloral hydrate - - - - - - | 2 " |
| Quinine (alkaloid) - - - - - - | 10 grains. |
| Carbolic acid (pure) - - - - - - | 30 " |
| Oil of lavender - - - - - - - | 10 drops, |

—MIX.

Generally, antiseptics can be used with good effect in many cases; but the disinfectants—those that can be used with safety—mostly fail to disinfect, so too much reliance should not be placed upon them. Suitable precautions should be taken to render their use unnecessarily; impure air should never be breathed, nor should preventable causes be allowed to pollute the air. Decay and decomposition can usually be prevented by antiseptics, if in no other way for it is much easier to prevent putrefaction from setting in than to check its progress after it has once begun.—*Ohio State Journal.*

